# Cyclic Bending Reliability and Failure Mechanism of Printed Biodegradable Flexible Supercapacitor on Polymer Substrate

**DOI:** 10.1021/acsami.2c08502

**Published:** 2022-08-23

**Authors:** Zhao Fu, Markus Hannula, Aarne Jauho, Kaisa-Leena Väisänen, Marja Välimäki, Jari Keskinen, Matti Mäntysalo

**Affiliations:** † Faculty of Information Technology and Communication Sciences, Tampere University, FI-33720 Tampere, Finland; ‡ Faculty of Medicine and Health Technology, Tampere University, FI-33520 Tampere, Finland; § VTT Technical Research Centre of Finland Ltd, Kaitoväylä 1, FIN-90571 Oulu, Finland

**Keywords:** flexible supercapacitor, printed electronics, PLA/barrier, cyclic bending reliability, failure mechanism

## Abstract

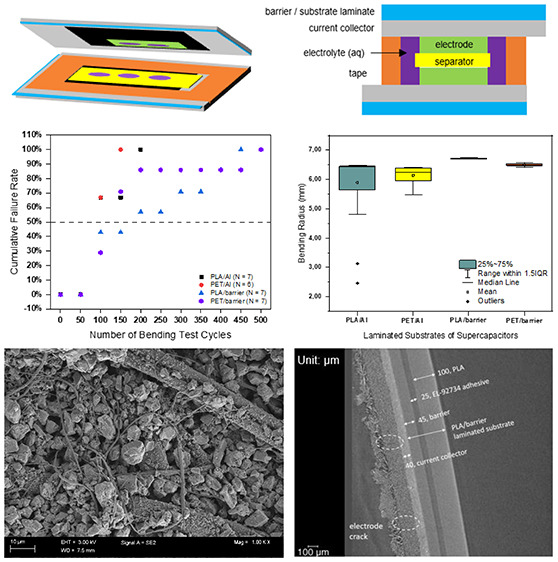

A flexible supercapacitor (SC) is an attractive energy storage device for powering low-power sensors, since it can be built using only nontoxic and sustainable materials. In this study, the advantages of using biodegradable polylactic acid (PLA) substrate for printed SC are investigated by studying the SC’s cyclic bending reliability, failure mechanism, and the impact of the bending radius. The results confirm that the SCs with laminated PLA with polymer barrier substrate exhibited the highest bending reliability, stability, and capability in preventing liquid electrolyte evaporation among the investigated substrates. Besides, the reliability decreased with the decreasing bending radius only when the strongly impacted areas lie on the electrode, the flaking and cracking of which was found to be the failure mechanisms of the tested SCs, except for the SCs with PLA/Al substrate, which failed due to the Al cracking. This research suggests that using PLA/barrier substrate, developing more robust activated carbon electrodes, developing cellulose paper with more dense fiber structure and smaller porous areas, and controlling the bending radius are crucial to improving the SC’s reliability.

## 1 Introduction

The Internet of Things has gained increasing concern for its potential to create a highly digital and efficient living environment. To reach this mission, the Trillion Sensors Initiative started in 2013, in the United States, which predicted that a trillion sensors would be used per year and that each of the current 7 billion people in the world would use around 140 sensors every year.^1^ To power these sensors, environmentally friendly and earth-abundant materials for energy storage devices, based on low-cost, energy-efficient production techniques, are in high demand. Among these devices, the supercapacitor (SC) has been increasingly considered for its advantages in providing high power density, long shelf life, quick charging capability, and good cycling stability.^2−4^ Furthermore, the recent developments in wireless sensors have decreased the required power down levels to sub-milliwatts enabling self-powered autonomous sensor nodes that harvest the required energy from the environment.^5^ Local energy storage is needed since the primary energy source such as light, thermal, or vibration is not continuously available, or the required peak power level of the application exceeds the level they can provide. However, to facilitate the application of an SC, sustainable materials and an energy-efficient fabrication method are crucial.

Printed electronics provide simple, low-cost, and energy- and material-efficient production methods for fabricating an SC with advantages in maximized energy density, applicability to a wide range of materials, and easy manufacturing of prototypes.^6^ For SC, thick films (on the order of tens of micrometers) of the active material are needed, whereas the print resolution is not so critical.^7,8^ Doctor-blade coating^3,9−12^ is an ideal fabrication method regarding these issues due to its simplicity, good control of the printed layer’s thickness, and low-temperature operation.

The alarming amount of the electronics waste (44.7 Mt worldwide in 2019^13^), especially plastics, which account for 27% (12.23 Mt, 15 million EUR) of all materials by weight and by value in 2016,^14^ has driven the researcher to seek biodegradable materials for electronic devices and components, as the full degradation of plastics takes 500 to 1000 years.^15^ A typical SC consists of substrate, current collectors, carbon-based electrodes, separator, and liquid electrolyte. To prevent the evaporation of electrolyte, a layer of metal oxide^16,17^ or aluminum^18−20^ has been commonly applied onto the substrate. Poly(ethylene terephthalate) (PET) film, aluminum layer, and their laminates have been used for printed flexible SC and have exhibited extended lifetimes.^3,9,21,22^ However, PET is not biodegradable.^23^ Biobased and industrially biodegradable polylactic acid (PLA)^23−25^ has been proposed to be a promising substrate alternative for flexible electronics, as it has exhibited excellent electrical and mechanical properties, and its main drawback of poor heat resistance and inherent brittleness can be improved via orientation and annealing treatments. In this research, the benefits of the high-heat PLA (hhPLA), with evaporated Al and laminated polymer barriers as the substrate for printed SC, are investigated.

Except the biodegradable materials and efficient fabrication method, the understanding of the failure mechanism^26^ and reliability^26,27^ of flexible SC, based on biodegradable material, is also crucial. However, SC technology is evolving, and the existing understanding of the SC failure mechanisms is still limited.^26^ The reliability studies on SCs have mainly focused on the electrical stability,^3,28,29^ whereas the mechanical reliability has not been investigated enough. For flexible devices, bending is a common deformation subjected in an application. Some cyclic bending tests^30−33^ have been applied together with electrochemical characterization to verify the robustness of the SC device. For example, Yeo et al.^32^ used a cyclic bending test to test a Ag nanoparticle film on the PET substrate, with one end moving and another end kept stationary. This method is suitable to test the highly flexible sample with low thickness. However, when there are different devices with dissimilar materials and stacking layers, the bending radii can be different even when the distance between the clamps is the same. In addition, the failure mechanism of the printed SC under cyclic bending has rarely been reported. Therefore, in this study, a cyclic bending test with verified repeatability and measurement accuracy is used to evaluate the reliability of the printed SCs with different substrates and to investigate the failure mechanism and the factors influencing the reliability of these SCs.

## 2 Experimental Section

### 2.1 Materials

The 125-μm-thick PET (Melinex ST506), 100-μm-thick hhPLA substrates, 15-μm-thick Al foil, and 50-μm-thick barrier layer were laminated to four types of laminates (PLA/Al, PET/Al, PLA/barrier, and PET/barrier), with 25-μm-thick EL-92734 adhesive, using Drytac JM26 tabletop laminator. The thicknesses of the four laminates were 130–145, 150–165, 160–170, and 170–175 μm, respectively. The Henkel PF407C graphite ink, activated carbon powder Kuraray YP-80F and chitosan binder Sigma-Aldrich 50494, aqueous NaCl (NaCl/H_2_O = 1:5) with 99.8% purity, and Dreamweaver Titanium 40 cellulose paper were used as current collector, electrode, electrolyte, and separator, respectively. The 3M 468MP-200MP adhesive tape was used for sealing the SC.

### 2.2 Supercapacitor Fabrication

**Figure 1:**
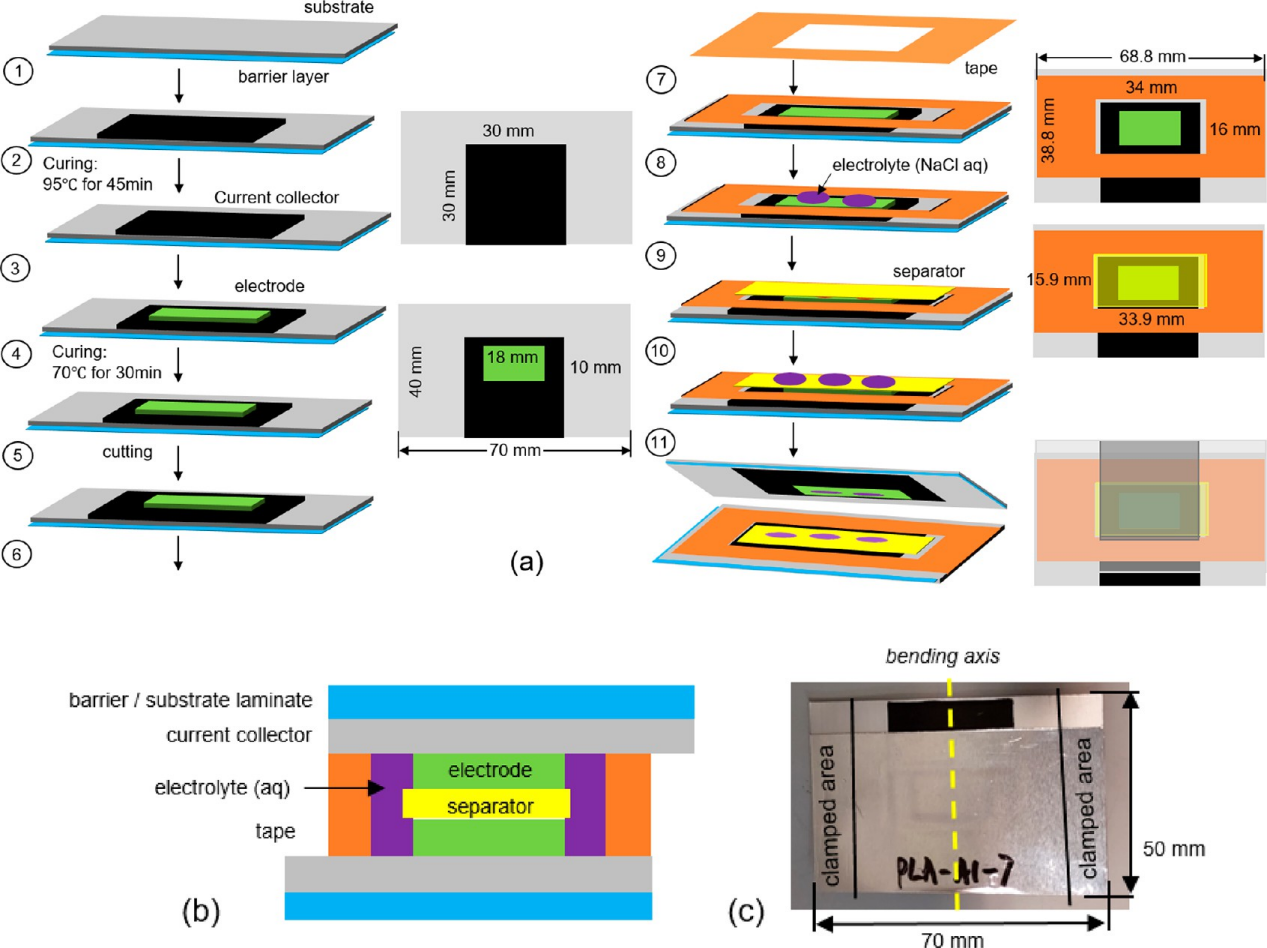
(a) Schematic illustration of fabrication procedure of printed flexible SC. (b) Schematic of printed flexible SC structure. (c) A sample of fabricated printed flexible SC.

The investigated SC samples were fabricated by the procedure presented in Figure 1a. The laminate was first cleaned using isopropanol, and two layers of 50-μm-thick polyimide (PI) films were cut to the identical size and location as the planned current collector in the substrate using a Silhouette Cameo Plus machine. The current collector layer was printed onto the laminate (step 1), and the electrode layers were printed onto the current collector layer (step 3), using doctor blade coating (Mtv messtechnik CX 4 motorized film applicator) with PI film masks. The wet thickness of both layers was 100 μm. The printed current collector and electrode were cured in the oven at 95 °C for 45 min and at 70 °C for 30 min, respectively. After cooling to room temperature, the thickness of the current collector and electrode layers were measured, and they were found to be 40–45 μm each. Then, the laminate was cut to the designed size (70 mm × 40 mm), using a Silhouette Cameo Plus machine. By then, half of the sandwich-structured SC was ready. Then, the prepared tape was attached around the electrode, as presented in the right-side illustration of step 7. Next, around 0.075 g of electrolyte was applied to cover the electrode (step 8). A 40-μm-thick separator was applied to the empty area of the tape (step 10). The electrolyte was applied to the separator and to the electrode of the other laminate, which was assembled to the first-half part of the SC sample (step 11). The assembly of components was assisted by a customized assembly and alignment tool manufactured with tough PLA by Ultimaker 3 3D Printer. To prevent the strong impact to the current collector, due to the small bending radius at the areas where the samples are clamped, a 10 mm-wide structural safe distance was designed to widen the substrate. The functionality of the samples was verified by measuring their initial electrical performance.

### 2.3 Cyclic Bending Test

The bending reliability of the printed SCs was evaluated by a cyclic bending test using an ESM 303 Mark-10 motorized tension test stand. The SC sample was first fixed by the clamps but without significant tension, which was followed by a height calibration and testing parameters setting. The lower testing grip was kept static, whereas the upper testing grip moved at a speed of 350 mm/min. The higher and lower limits were set to define the travel trajectory of the upper grip; the lower limit determines to what extent the flexible SC would be bent, which accurately corresponds to the bending radius as reported in ref (34). The SC’s mechanical change and failure were observed visually during the tests. The bending test and the SC’s electrical performance characterization were conducted in 50 cycle intervals, until the electrical failure was reached. To investigate the evolution of electrical properties of biodegradable devices, the SCs with a PLA/barrier laminate were further tested under different bending distances until aggressive failure was shown.

### 2.4 Bending Radius Measurement

The samples’ bending radii were measured using camera imaging and the proportion calculation method. When the upper grip moved to the position of low limit in the first cycle, the sample was subjected to the strongest impact and bent to its smallest radius; it was imaged by a Canon G11 camera, which was held by a tripod, and the height and focusing were adjusted for optimal imaging quality. All images were taken with the same magnification. The image was measured using Inkscape software. A circle was fit to the bent round shape to determine the bending diameter. The ratio of the drawn circle diameter *d* (in pixel) and the distance between upper and lower grips *h* (in pixel) is used to calculate the bending radius *r* (in mm), since the real distance between the upper and lower grips *H* is known. Thus, the real bending *r* (in mm) of the sample can be computed by the following equation.
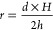
1

### 2.5 Supercapacitor Characterization

The SC samples’ electrical performance before and after each test was measured by a Maccor 4300 device, with 1200 mV charging and discharging potential. The positive probes and negative probes were connected to two sides of the SC the identical way in each measurement. The sample was first charged and discharged with a constant current up to 1.2 V three times, and then the voltage was kept at 1.2 V for 30 min and discharged with a constant current. The capacitance was defined during the constant current discharge step between 0.96 and 0.48 V potential. The leakage current of the SCs was determined with a float current experiment: the capacitor was charged to 1.2 V, and the current was recorded after holding that potential for 1 h.^3^ By standard IEC 62391–1, equivalent series resistance (ESR) is defined from the IR drop, when the constant current discharge is initiated. The IR drop is defined from the crossing point of the linear regression at the beginning of the discharge curve and the time point when the discharge is initiated. In this research, the focus was on capacitance, ESR, and leakage current. The SCs’ mass change was monitored by mass measurement, which was performed using the Fisher Scientific PAS214C Analytical Balance scale, with a resolution level of 0.1 mg.

### 2.6 Failure Analysis

The electrically failed samples were imaged by a Zeiss Xradia MicroXCT-400 (Zeiss) microtomography (μ-CT) device, which takes 1201 X-ray projections from the full 360° of the rotation angle. They were used to construct the three-dimensional (3D) volume using Zeiss XMReconstructor software. Two magnifications were used for imaging—a general overview image of the sample with a 22.6 μm pixel size and a more accurate image with a 5.2 μm pixel size. The image processing and visualizations were done with the Avizo 2020.2 software (Thermo Fisher Scientific). The material density difference appears in the image, which indicates changes in material thickness and profile.

The separator papers were taken out using a laboratory scalpel; its electrical conduction was verified using Keithley 2425 multimeter. The surface morphology of the separator paper and carbon powder samples was studied by a field-emission scanning electron microscope (FESEM, Zeiss ULTRAPlus, Carl Zeiss Microscopy GmbH) at the Tampere Microscopy Center, Tampere University. The secondary electron detector mode, with an acceleration voltage of 3.00 kV and an aperture size of 30.00 μm, was used for scanning electron microscopy (SEM) imaging. The separator samples were prepared by attaching the paper samples on aluminum pin stubs, using adhesive conductive carbon tabs. The active carbon powder was sprinkled directly onto an adhesive carbon tab. The samples were coated with 2 nm Pt/Pd alloy (80/20), using a high-vacuum sputter coater (Leica ACE600, Leica Microsystems CMS GmbH).

## 3 Results and Discussion

### 3.1 Mass Loss of SCs with Al Substrate Layer

**Figure 2:**
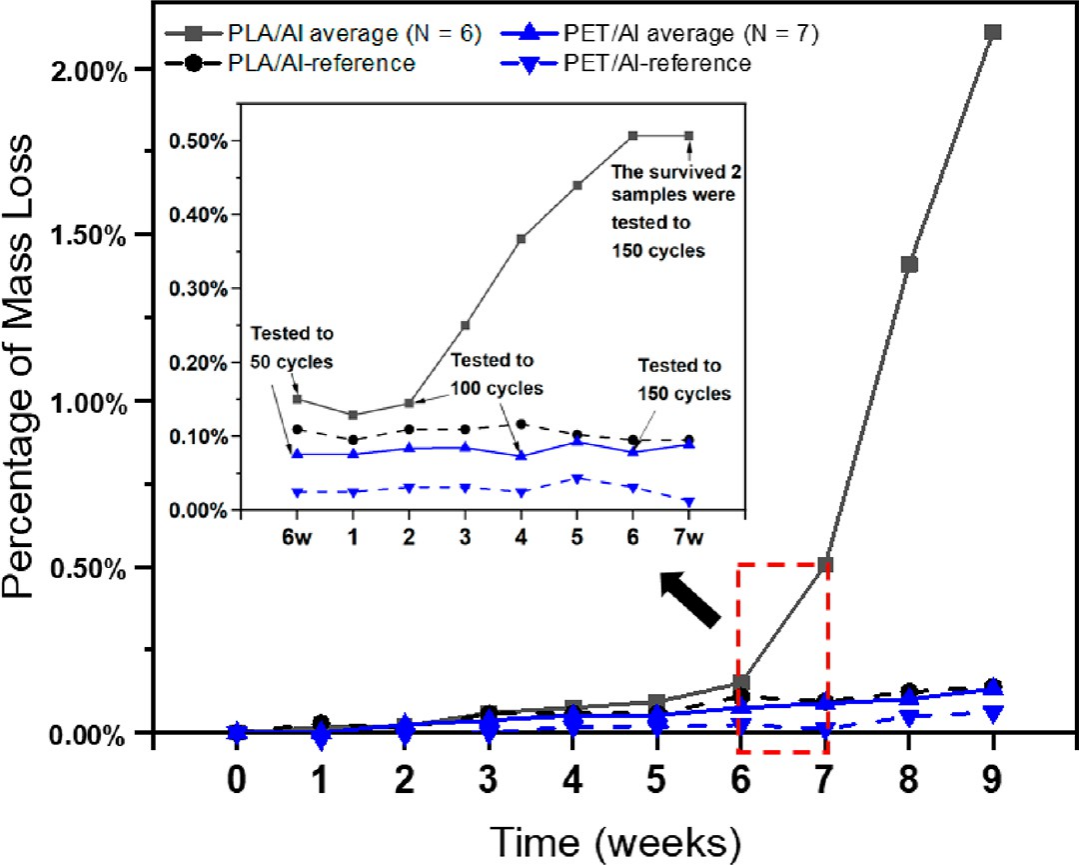
Mass loss of the supercapacitor samples with PLA/Al and PET/Al substrates.

The seven SC samples, with both PLA/Al and PET/Al laminated substrates, were first subjected to 10 cycles of a bending test, during which all samples exhibited Al cracking. Then, the mass of them and reference samples (not tested) were measured to investigate if the Al cracking would cause electrolyte evaporation. After six weeks, the mass of the bent SCs with PLA/Al substrate had decreased around 0.15%, while the reference sample PET/Al had a mass loss of 0.11%. The SCs with PET/Al substrate lost around 0.08% mass, which is also slightly higher than the mass loss of reference sample PET/Al, which is 0.03%. It can be concluded that the mass losses of damaged samples were slightly higher than corresponding reference samples but certainly not dramatic. Furthermore, the mass loss of the SC with a PLA substrate was slightly higher than PET due to a higher water vapor permeation rate of PLA than PET.^35^ It also worth mentioning that the sealing can also cause minor mass loss, as extremely small amounts of mass losses of the reference samples were observed. Keskinen et al.^9^ reported that tight sealing is needed to keep dioxygen out of the flexible SC, as a possible cause of self-discharge was stressed. However, the impact of the sealing thickness on the mass loss requires further investigation. In this research, the mass of electrolyte in each SC sample was around 75 mg. After six weeks, the SCs lost around 2 mg of mass. By estimation, the lost electrolyte through evaporation takes up less than 3% of the electrolyte, which is still an extremely low level of mass loss. After six weeks, six out of seven samples were then tested with 40 more cycles followed by an electrical performance measurement, and one sample was not tested further and was instead used as a reference. After this, 50 more cycles of the bending test and subsequent electrical performance measurement were conducted if the electrical failure criteria had not been reached, which are described in Table 1. Such test and measurement cycles were repeated until one of the failure criteria was reached. It was found that the mass of the SCs with PLA/Al decreased dramatically after the samples electrically failed after 100 or 150 cycles of the bending test. Their mass had lost, on average, 2.11% after nine weeks, whereas the SCs with PET/Al substrate had lost only 0.13% in the same period of time, which is close to the 0.06% mass loss of the PET/Al reference sample. The details are presented in Figure 2. This indicates that the SCs with a PLA/Al substrate experienced significant electrolyte evaporation through the cracked Al layer, whereas the electrolyte evaporation of SCs with PET/Al substrate was negligible. This is also confirmed by failure images of Figure 7a,c. Dogre et al.^36^ also reported that the flexibility of an SC is limited by the Al foil substrate in the cyclic bending test, since cracking^37^ has been a limitation when Al is used as a barrier layer in the laminate.

**Table 1:** Failure Criteria Applied in the Research

failure category	parameter/component	failure criteria	refs
electrical performance	capacitance	20% decrease	(3 and 38−42)
	ESR^a^	100% increase	(38−40)
	leakage current	100% increase	(43)
mechanical performance	substrate	cracking	
	graphite ink	delamination	

a Note: ESR is the abbreviation of Equivalent Series Resistance.

### 3.2 Electrical Properties Evolution & Reliability of SCs with Different Substrates

During the six weeks of monitoring the SCs with PLA/Al and PET/Al substrates and reference samples, their electrical performance was measured weekly. The capacitance of the SCs with PLA/Al substrate and reference samples decreased similarly and steadily with time; a decrease of around 3% in six weeks was detected. The SCs with PET/Al substrate lost around 4% capacitance by six weeks. The ESR of the samples and reference samples exhibited a similar and slight decrease of around 0.7 Ω in six weeks. The change in leakage current in the six weeks was also within 0.5 μA for all samples. These changes are not significant if the measurement error is taken into consideration.

**Figure 3:**
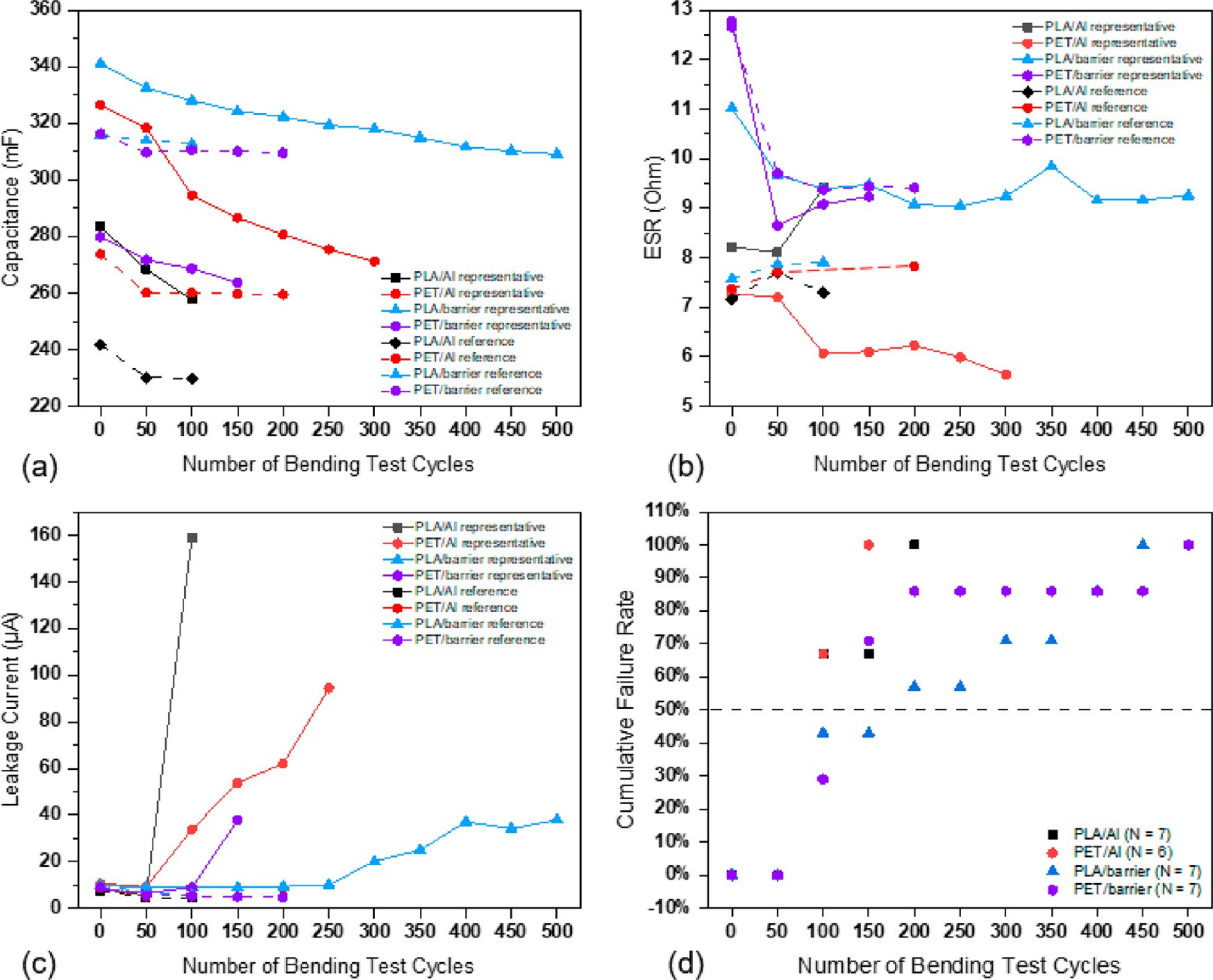
Evolution of electrical properties: (a) capacitance, (b) ESR, and (c) leakage current, with the number of bending test cycles of representative and nontested reference supercapacitor with different substrates, and (d) their cumulative failure rates (Note: a representative sample fits the accumulative 50%, which represents the most middle case of all duplicated samples).

To further investigate the evolution of the electrical performance as a function of the number of test cycles, the samples were tested more, in 50 cycles intervals. The results of representative samples and nontested reference samples PET/Al and PLA/Al are presented in Figure 3a–c. The SCs with PLA/Al substrate failed early due to the sudden and sharp Al cracking, while their capacitance had not decreased significantly. The SCs with PET/Al substrate had lost 14% capacitance on average after 250 cycles of the bending test, which is slightly higher than that of the PET/Al reference sample. In comparison, the SCs with a barrier layer experienced slower and less capacitance loss of up to 10–12% after 500 cycles of the bending test. In comparison, the reference samples had lost 5–15 mF (2–4%) capacitance generally, which is within a reasonable range regarding the environmental influence and aging. For the tested SCs, with the evaporation of water from the electrolyte, part of the carbon surface may lose contact with the electrode, which makes them not functional, thus decreasing capacitance.^9^ Therefore, the higher level of capacitance loss of the SCs with the PET/Al substrate than that of the SCs with barrier layer reveals that the barrier layer exhibited a higher level prevention of electrolyte evaporation than the Al layer. The SC with PLA/Al did not exhibit such a high portion of capacitance loss, as its test ended early due to the sudden mechanical cracking of substrate. However, characterizing it without causing extra damage to the sample remains a challenge.

The ESRs of most samples were stable with variation within 2 Ω, whereas the ESR of SCs with a PET/barrier substrate decreased around 4 Ω after 50 cycles of the test, which is similar to the change of ESR of its non-tested reference sample, as presented in Figure 3b. The error in the measurement and the impact of environment can be the causes of the variation of the ESR value. The sharp cracking of the PLA/Al substrate probably also caused the cracking of the current collector, which was seen in a few samples. In the case of the SC structure, ESR has been found to mainly depend on the current collector dimensions and materials.^9^ The materials and original structure of the carbon collector of these four types of SCs are the same, whereas the Al cracking can cause the failure of the current collector, which can cause significant change to the ESR value.

The leakage current of the SCs with PET/barrier and PLA/barrier substrates exhibited a similar and slower increase with the number of test cycle, whereas the SCs with an Al substrate layer experienced a more aggressive and rapid increase in the leakage current. This may partly be due to the Al cracking, which led to the entry of oxygen. It can dissolve into the electrolyte and be adsorbed onto the surface of activated carbon.^44^ It has been reported that the leakage current is most likely due to the Faradaic reactions of impurities in the SCs.^9,28,45^ Besides, after the loss of material due to the repeated bending impact, the electrodes may be in contact and lead to the increase of the leakage current. These explain the dramatic increase of the leakage current of SCs with PET/Al and PLA/Al substrates. In comparison, the leakage current of the non-tested reference samples did not show a noticeable change.

Overall, the SCs with a PLA/barrier substrate exhibited the most steady change in electrical properties, which make the failure occur more slowly and with higher reliability. In comparison, the SCs with a PET/Al substrate exhibited the most dramatic change in electrical properties, which caused a rapid failure and lower reliability. This issue is also confirmed by the cumulative failure rates of the SCs with different substrates, as presented in Figure 3d. The cyclic bending reliability of the SCs with different substrates follow a sequence of PLA/barrier > PET/barrier > PLA/Al > PET/Al by a 50% cumulative failure rate. Thus, the SCs with a polymer barrier layer exhibited significantly higher reliability than the SCs with an aluminum layer. Bichler et al.^46^ reported that using several layers in a laminate increases the barrier effects because of the properties of each layer and the reduced stressing effects. Coating metals or some oxides onto polymer foils, especially like PET, was also found to improve impermeability to water vapor significantly.^37,46^ In this research, the barrier layer (3M FTB 3–50) has a multilayer structure, consisting of PET and metal oxide, which provides high impermeability to water vapor. In comparison, Al foil is subjected to flex cracking due to the tensile stress as reported by Lamberti et al.^37^ During the bending test, the Al foil cracked almost immediately (within 10 cycles), which caused concentrated stress and led to the failure of SCs.

### 3.3 Impact of SC Substrate on Bending Radius Stability

**Figure 4:**
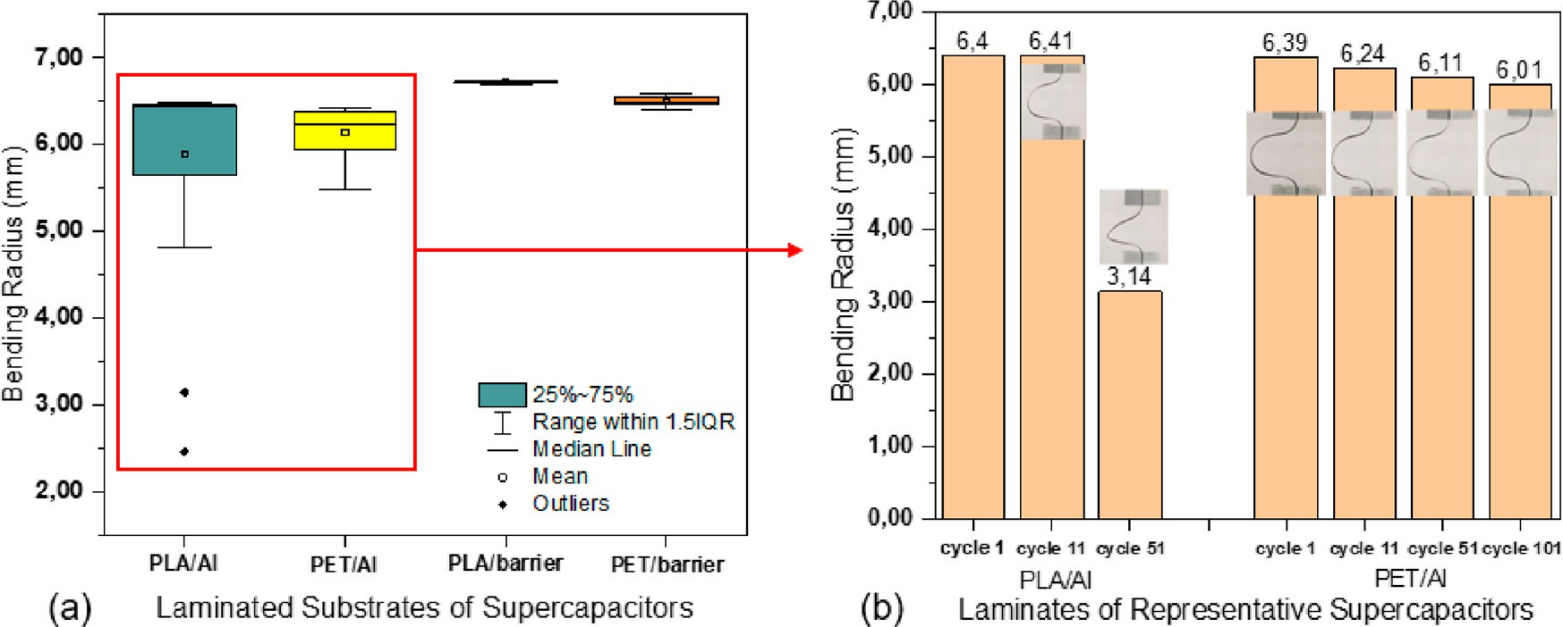
(a) Bending radius of supercapacitor samples (*N* = 7) with different substrates and (b) different evolution of bending radius of SCs with aluminum substrate layer.

The bending radii of the SCs were measured at the first cycle of each bending test; the results are presented in Figure 4a. The SCs with a barrier layer exhibited significantly higher stability in the bending radius along with the number of bending test cycles than the SCs with an Al layer, especially the SCs with a PLA/barrier substrate exhibited the smallest variation (0.3 mm) in the bending radius. The SCs with the PLA/Al substrate exhibit the largest variation in the bending radius, which is due to the sudden and sharp breaking of the Al, as presented in Figure 4b. Mehmood et al.^47^ studied the deformation of Al foil laminated onto polymer and observed that debonding in Al foil in the laminate caused the strain localization in an early stage during deformation, making it susceptible to breakage. This mechanism has been described by Dietmar and Thomson.^48^ Some researchers^49−51^ have also reported the fatigue crack initiation due to repeated loading. Besides, in the PLA/Al laminate, the adhesion between Al and PLA is good, which results in high stiffness and low toughness.^47^ This property makes cracking prone to occur when subjected to bending. In comparison, the SCs with the PET/Al substrate did not exhibit such a sharp decrease in the bending radius but experienced a more gradual decrease process.

### 3.4 Impact of Bending Radius on Mass Loss

The cyclic bending test of the SCs with different substrates confirmed that the SCs with a PLA/barrier substrate exhibited the highest reliability and the smallest variation in bending radius against cyclic bending, and it also prevented electrolyte evaporation well. Thus, the SCs with a PLA/barrier substrate were selected for the cyclic bending test, when the bending distance *H* was 30, 25, 20, and 15 mm, respectively. *H* refers to the distance between the upper and lower clamps when the SC is bent to its minimum curvature and subjected to the strongest impact. Seven SCs were tested with each bending distance, and they had been weighted weekly to investigate the impact of the bending radius on mass loss and electrolyte evaporation of the SCs. The SCs tested, when *H* = 30 mm, did not exhibit more mass loss compared with the reference samples. The SCs tested under other conditions exhibited a very low level of mass loss, which ranges between 0.12% and 0.25% loss of original mass on average. In addition, the samples tested in different conditions, except the case of *H* = 30 mm, had the major mass loss in the first one to two weeks, after which the mass was kept stable. These indicate that the bending with different bending radii did not cause significant mass loss to the SCs with PLA/barrier substrate, which, in a way, also verified the very low water vapor permeability of the PLA/barrier substrate. The PLA laminate has been widely reported to lower the water vapor permeability.^52,53^

### 3.5 Impact of Bending Radius on Electrical Properties Evolution and Failure

**Figure 5:**
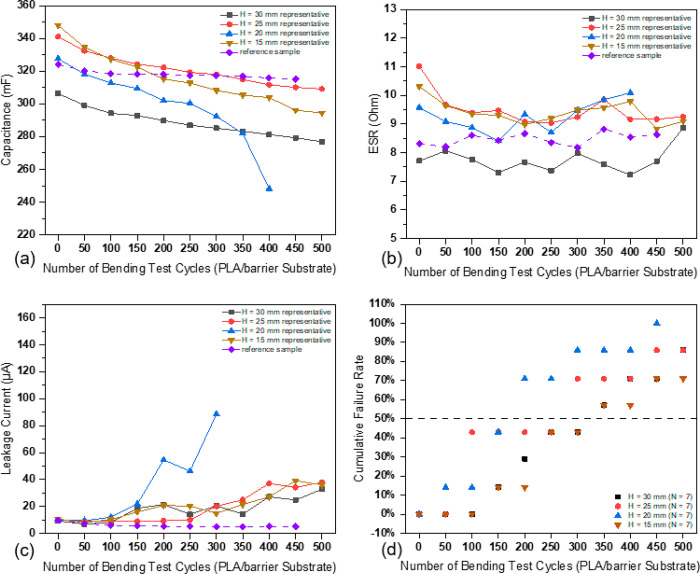
(a–c) Evolution of electrical properties as a function of the number of cyclic bending test cycle (Note: a representative sample fits the accumulative 50%, which represents the most middle case of all duplicated samples). (d) Cumulative failure rates of SCs with a PLA/barrier substrate (*H* = 30 mm refers to the condition when the sample is bent to its smallest curvature, the distance between the upper and lower clamps is 30 mm. The same for other values).

The evolution of electrical properties of SCs with a PLA/barrier substrate tested under different bending radii as a function of the bending test cycle was investigated. The capacitance of the SCs tested in different conditions decreased similarly and steadily, with 10–15% capacitance lost after 500 test cycles, as presented in Figure 5a. The loss of capacitance can be caused by some issues. One is that the evaporation of water from electrolytes made part of the carbon surface lose contact with electrolyte and lose functionality.^9^ However, this reason is minor, since the PLA/barrier exhibited good protection of electrolyte evaporation. The other reason is that the repeated bending may have caused part of the electrodes to lose functionality, thus reducing surface area and losing capacitance as the capacitance is basically proportional to the surface area.^54^ It is worth considering that, for long-term use of the SC, impurities like oxygen can enter and affect aging characteristics through an oxidation/reduction reaction.^55^

During the 500 cycles of the bending test, the ESRs of the SCs remained stable with a small fluctuation that can be caused due to the measurement system. ESR was found to mainly depend on the current collector structure and materials,^9^ whereas all the SCs tested under different bending radii have the same current collector in structure and material. The representative cases of the leakage current change during the cyclic bending test, under each test condition, are presented in Figure 5c. The leakage current increased slowly during the first 150 cycles test, after which it increased faster, especially the SCs tested with *H* = 20 mm. The dramatic increase of leakage current can be due to the repeated bending, which may have loosened the active carbon particles from the electrodes. The separator paper between the electrodes was found broken under repeated bending impact, as shown in the images of Figure 7. The carbon particles can move and form paths for short circuits between electrodes.

**Figure 6:**
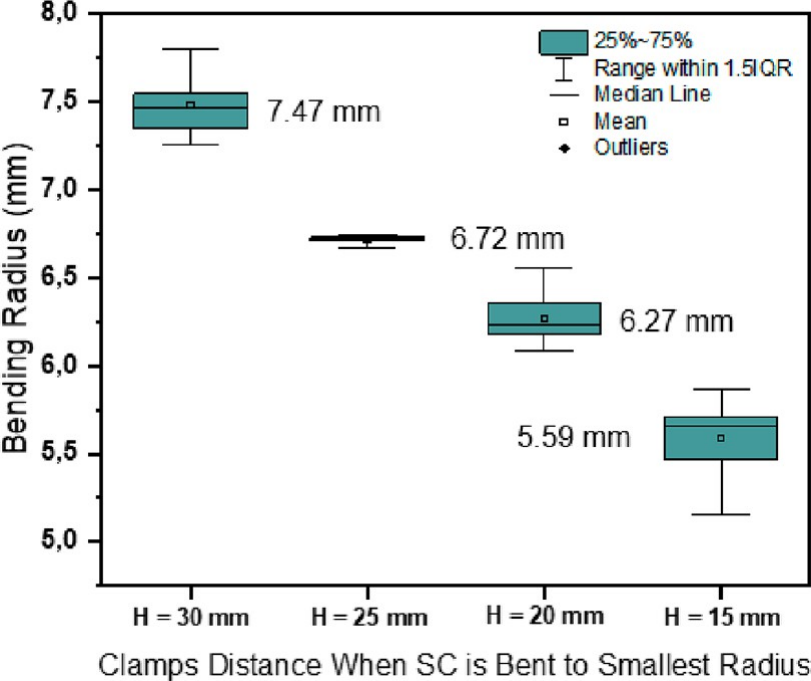
Bending radii of SCs with PLA/barrier substrate under different test conditions (*N* = 7 for each type of SC sample).

**Figure 7:**
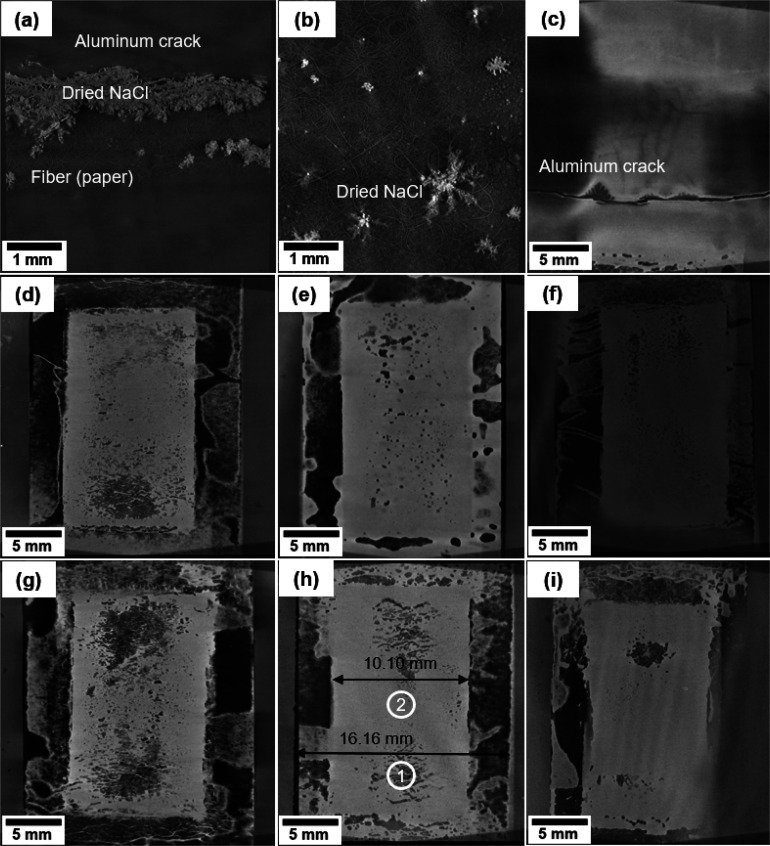
μ-CT images of representative SC samples with (a, b) PLA/Al substrate, (c) PET/Al substrate, (d) PET/barrier substrate, tested when *H* = 25 mm; (e) non-tested reference SC sample with PET/barrier substrate; and SC samples with PLA/barrier substrate tested when *H* = (f) 30, (g) 25, (h) 20, and (i) 15 mm.

Since all the samples in the bending test failed due to the increase in leakage current, the cumulative failure rate based on the failure in the leakage current was plotted, and the median failure point is used to characterize the failure of the SCs, as presented in Figure 5d. The results show that the cyclic bending reliability of the tested SCs follows the relation of (*H* = 30 mm) = (*H* = 15 mm) > (*H* = 25 mm) > (*H* = 20 mm), which was verified by the μ-CT failure images in Figure 7. The bending radii of the SCs were measured at the first cycle of the bending of each test, and the results are presented in Figure 6. The bending radii of the SCs tested under different conditions show small and similar levels of variation (at a range of 0.3 mm), especially when *H* = 25 mm, the bending radius exhibited the highest stability. The results indicate that the bending radius of the SCs with a PLA/barrier substrate was quite stable, and it is not clearly dependent on the bending impact nor the bending radius.

### 3.6 Failure Analysis

#### Failure Phenomena of Different SCs

The representative samples of the tested and failed SCs and the reference samples were imaged to investigate the locations and mechanisms of failure. By the working principle of X-ray μ-CT imaging, the darker areas in the images indicate the lower density of the material than the brighter areas. The SCs with PLA/Al and PET/Al substrates all exhibited Al cracking in the first 10 cycles of the bending test, and the cracking became more severe in further tests. However, the difference is that the Al cracking of the SCs with a PLA/Al substrate led to the aggressive evaporation of liquid electrolyte, which dried and accumulated at the crack area, as presented in Figure 7a. The dried NaCl electrolyte was also commonly found on the surface of the separator paper, as the snowflake-shaped bright parts, shown in Figure 7b. However, the Al cracking of the PET/Al substrate did not lead to such significant leakage of electrolyte, as presented in Figure 7c. In all the tested SCs, except the ones with a PLA/Al barrier, the two sides of the separator and electrode generally exhibit lower density, as the dark areas show. In comparison, the middle area of the sample is bright. The SC with a PET/barrier substrate presented in Figure 7d is an example. By contrast, the reference samples with different substrates only exhibited small dark spots across the sample, as presented in Figure 7e. The lower density of the spot areas in the non-tested reference samples can be caused by the excessive amount of liquid electrolyte and the entry of air during sample fabrication. The lower-density areas in the SCs with a PLA/barrier substrate also lie to the two sides of the separator and electrode, as presented in Figure 7f–i. However, the size of the dark areas of the SCs tested under different conditions differs. The SCs tested when *H* = 25 mm and 20 mm show similar and significantly larger dark areas than the SCs tested when *H* = 30 and 15 mm. There are some exceptionally large areas with prominent levels of darkness in many images, which are due to the lower thickness of the areas rather than any failure. The dimension in Figure 7h shows that the layer in the middle is the electrode, as its width is measured to be 10.10 mm, and the layer marked with 16.16 mm width is the separator. Thus, at the left and right sides, there are hollow areas between the electrode and separator, which cause the lower density of the material, showing large and very dark areas in the image. In addition, no graphite ink failure was present in any tested sample.

#### Failure Mechanisms of Electrode

**Figure 8:**
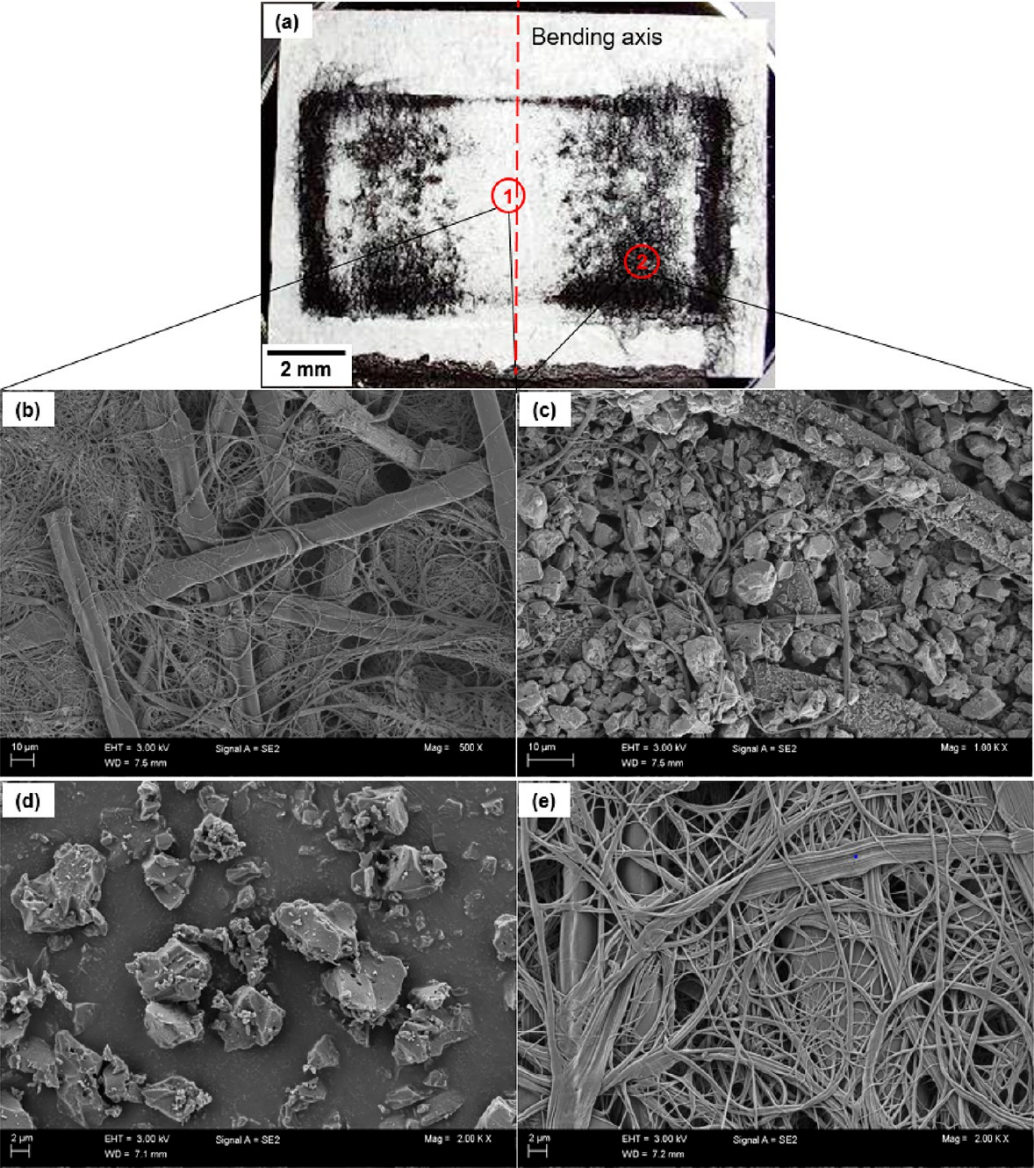
(a) Optical image of separator paper; FE-SEM images of (b) the separator of area 1 marked in image (a); (c) the separator of area 2 marked in image (a); (d) the particles of Kuraray YP-80F activated carbon; (e) the fiber structure of a reference Dreamweaver Titanium 40 cellulose paper (separator).

To further investigate the mechanisms of the formation of the dark areas, the separators of some representative SCs were taken out for electrical characterization and SEM imaging. A large number of black particles was on the separator, as presented in Figure 8a, and its electrical conduction was confirmed by using a Keithley 2425 multimeter. The FE-SEM images prove that, in the dark areas of the μ-CT images, the fiber structure of the separator paper is full of active carbon particles (Figure 8b), and the darker the area, the thicker the carbon particle layer. By contrast, such carbon particles area rarely found in the light areas of the μ-CT images (Figure 8c). This indicates that, in such dark areas, the repeated bending test had caused the flaking of carbon particles from the electrode layer, and they were moved into the porous areas in the fiber structure of the separator. To further verify the possibility of carbon particle migration into the fiber structure, the Kuraray YP-80F activated carbon (Figure 8d) and the fiber structure of the separator (Figure 8e) were imaged. The size of its particles is found to be 0.5–5 μm, and the size of the porous areas of the fiber structure of the separator is up to 4 μm. The manufacturer^56^ and Arvani et al.^57^ reported comparable results, claiming that the porous structures have a size up to around 3 μm. Therefore, a large number of carbon particles from the electrode are doubtlessly able to enter the porous areas of the fiber structure of the separator, which can also form the electrical conduction paths. This explains the increased leakage current. The flaking of electrode particles is shown in the loss of capacitance of all tested SCs. In addition, no cracking of the fiber structure or interface delamination was observed in different SCs.

#### Failure Mechanisms of All Components

**Figure 9:**
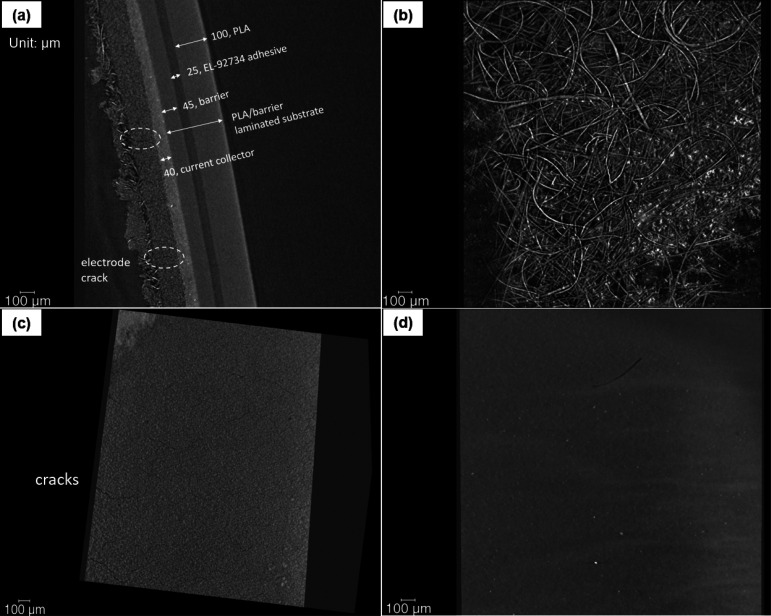
μ-CT images of (a) the structure of SC samples; (b) separator; (c) 3D imaging of electrode layer; (d) 3D imaging of current collector later. (Note: image (a) has orientation, which makes the scale bar 100 μm look shorter than the 100 μm thick PLA).

The density of the stacked material can be influenced by separator, electrode, and current collector. To further investigate if there were any failures in each layer, the 3D imaging was conducted using a μ-CT device. The components of the SC were verified by their thickness, as presented in Figure 9a. No interface delamination or crack was found in the failed samples. Besides, the μ-CT imaging enabled us to observe any layer of the sample in 3D mode, which also did not find any interfacial failure. No cracking was found in the fiber structure of the separator (Figure 9b), which agrees with the FE-SEM imaging results. The cracks in the electrode are commonly seen as shown in Figure 9a,c, whereas no crack was found in the current collector.

#### Severeness of Impact across the Electrode

Figure 8a shows that the carbon particles are densely distributed on the separator, where the short edges and the corners of the electrode lie. This indicates that the edges and sharp corners of the electrode have flaked more particles than other areas. Strain concentration at the edge and corner areas has been commonly understood by both experiments and finite element simulation.^58,59^ In addition, the short edges of the electrode layer in the SCs were also in the areas subjected to high impact during the repeated bending by analyzing the geometry under bending conditions. This can be further confirmed if the simulation work can be conducted to visualize the strain distribution.

In addition, the bending condition also influenced the severeness of the electrode particle flaking. The results of the reliability test based on the electrical failure criteria in Figure 5d reveal that the SCs tested when *H* = 25 and 20 mm failed significantly sooner than the SCs tested when *H* = 30 and 15 mm. When *H* = 30 mm, the sample had not been bent extensively, and the impact was relatively light. When *H* = 25 and 20 mm, the two areas close to the ends of the electrode were subjected to the strongest impact due to the small bending radius there. However, when *H* = 15 mm, the highly impacted area might have moved outside the electrode when the SC was bent to such a small radius, the confirmation of which can be included in future work.

### 3.7 Discussion on Issues for Improving SC Reliability

Except for the SCs with PLA/Al substrate, which failed very soon due to the Al cracking, the tested SCs were confirmed to fail due to the electrode cracking and the flaking of carbon particles from electrode, which enter the porous areas of the fiber structure of the separator and form an electrical conduction path. To improve the SC reliability, two approaches are crucial. One is to mechanically develop more robust activated carbon electrodes to reduce flaking; the other is to develop cellulose paper with a more dense fiber structure and smaller porous areas to limit the migration of carbon particles into it. Increasing the thickness of the separator paper in the SC would also reduce the probability of forming an electric conduction path through the separator. In addition, the sharp edges and corners were found to lose most carbon particles due to the high impact over those areas and due to the mechanical strain concentration. Designing the electrode layer with a round shape and large curvature, instead of a right angle, can eliminate the local strain concentration.

This study has revealed some factors that have limited the reliability of SCs. First, in sample fabrication, the entry of the impurities and oxygen can cause leakage current and self-discharging through a Faradaic charge-transfer reaction. Vacuum treatment of the electrodes, prior to assembly and bubbling the electrolyte with inert gas, are considered to reduce the amount of absorbed oxygen.^9^ Second, the thickness distribution of the SC is uneven across the SC, as overlapping current collectors, electrodes, and the separator make the middle area thicker than the edges of the SC. This is also the main source of variation in the calculated bending radius, as it caused the SCs to not have the standard circular shape but a slightly tilted shape when subjected to bending. To overcome the uneven thickness distribution, filling the thickness gap of the edge areas with sealing adhesive is recommended to explore.

## 4 Conclusions

In this research, the cyclic bending reliability of the SCs fabricated by doctor blade coating with PLA/Al, PLA/barrier, PET/Al, and PET/Al laminated substrates was investigated. The SCs’ mass and electrical performance (capacitance, ESR, leakage current) were characterized before and after tests. The SCs with PLA/barrier substrate exhibited the highest cyclic bending reliability and mechanical stability in bending radius during the test. Then, the impact of bending radius on the reliability, electrical property evolution, and failure mechanisms of the SCs with PLA/barrier substrate was investigated. The failure analysis was conducted using SEM and X-ray μ-CT. It was found that the SCs with a barrier layer exhibited significantly higher reliability than the SCs with PLA/Al substrate, which failed to the mechanical cracking of Al. The SCs with other substrates failed due to the flaking of carbon particles from electrode and the cracking of electrode in the two ends of the electrode. The flaked carbon particles entered the porous areas of the separator’s fiber structure and formed electrical conduction paths, which led to the significant increase in leakage current. No failure was found in interface delamination, current collector, nor fiber structure breaking of the cellulose paper (separator). This research suggests that using PLA/barrier substrate, developing more robust activated carbon electrodes, developing cellulose paper with more dense fiber structure and smaller porous areas, and controlling the bending radius are crucial approaches to improving the SC’s reliability. A simulation and modeling method can be used for further research in mapping the strain distribution across the SC during the cyclic bending.

## Notes

Data Availability. The data generated and analyzed during this research is available from the corresponding authors upon reasonable request.
